# Correction to “Salvage treatment after covalent BTKi failure: An unmet need in clinical practice in Waldenstrom macroglobulinemia”

**DOI:** 10.1002/hem3.70219

**Published:** 2025-09-23

Frustaci AM, Zappaterra A, Galitzia A, et al. Salvage treatment after covalent BTKi failure: An unmet need in clinical practice in Waldenstrom macroglobulinemia. *HemaSphere*. 2025;9(2):e70094. doi:10.1002/hem3.70094

In the Funding section, the funder was incorrectly listed. The funder statement now reads: “Open access publishing facilitated by Azienda Socio Sanitaria Territoriale Grande Ospedale Metropolitano Niguarda, as part of the Wiley – SBBL agreement.”

The original article has been updated. We apologize for this error.

